# Supplementary material for: Splice-Junction-Based Mapping of Alternative Isoforms in the Human Proteome
Source: Cell Rep. Author manuscript; Available in PMC 2020 Jan 15. (PMC6961840; doi:10.1016/j.celrep.2019.11.026)
Supplement: 2 [file NIHMS1546469-supplement-2.zip › DF1/PXD010154/Colon/Colon_2_EMSY_EGVSCSDEDEKPR.pdf]

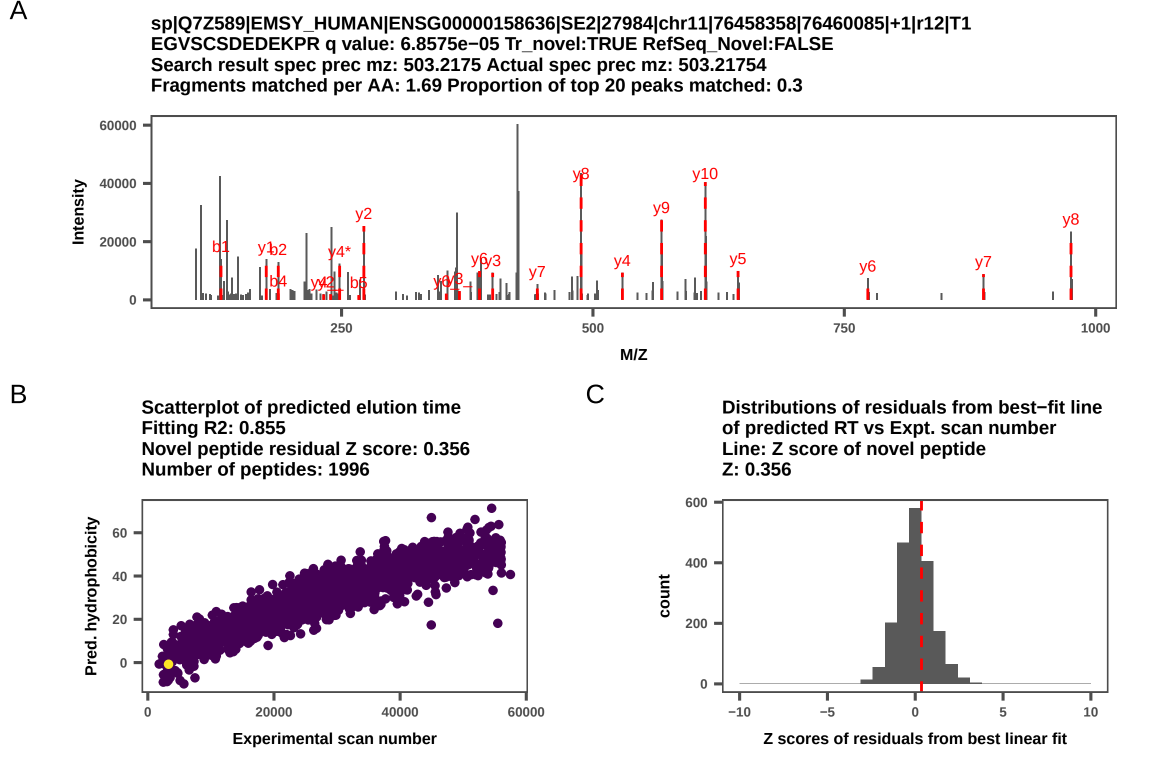
A
sp|Q7Z589|EMSY_HUMAN|ENSG00000158636|SE2|27984|chr11|76458358|76460085|+1|r12|T1
EGVSCSDEDEKPR q value: 6.8575e−05 Tr_novel:TRUE RefSeq_Novel:FALSE
Search result spec prec mz: 503.2175 Actual spec prec mz: 503.21754
Fragments matched per AA: 1.69 Proportion of top 20 peaks matched: 0.3
Intensity
60000
40000
20000
0
b1
y1
b2
b4
y2
y4*
b5
y6
y3
y7
y8
y4
y9
y10
y5
y6
y7
y8
250
500
750
1000
M/Z
B
Scatterplot of predicted elution time
Fitting R2: 0.855
Novel peptide residual Z score: 0.356
Number of peptides: 1996
Pred. hydrophobicity
60
40
20
0
0
20000
40000
60000
Experimental scan number
C
Distributions of residuals from best−fit line
of predicted RT vs Expt. scan number
Line: Z score of novel peptide
Z: 0.356
count
600
400
200
0
−10
−5
0
5
10
Z scores of residuals from best linear fit
